# Why Do We Need To Diversify the Microbial Sciences?

**DOI:** 10.1128/mSphere.00625-21

**Published:** 2021-07-21

**Authors:** Alfredo G. Torres

**Affiliations:** a University of Texas Medical Branch, Department of Microbiology and Immunology, Galveston, Texas, USA

**Keywords:** diversity, inclusion

## Abstract

We can learn from the diversity of microbial communities, represented by the thriving of different species. Failure to build inclusive microbial sciences only perpetuates an imbalance that is damaging to the scientific community. As microbiologists, are we ready to lead the way on diversity and inclusion initiatives and set an example for the rest of the professions and scientific fields?

## COMMENTARY

Working in the microbial sciences has led investigators to appreciate that successful microbial communities are diverse in their species composition, resulting in benefits to all their members while they reach an effective balance (homeostatic state). Disruption of the diversity of species due to the attack of a pathogen or the variability in environmental conditions can generate an imbalance (dysbiosis), resulting in damage or disease.

Over the last year, many organizations around the nation, including those involved in higher education and societies, such as the American Society for Microbiology (ASM), began to have productive conversations to address the inequities prevalent in science and academia. Working in the microbial sciences, where diversity is a key element in finding a homeostatic balance, has led Beronda Montgomery and other microbiologists to pioneer the idea that specific actions have to be implemented to address pervasive practices that prevent colleagues from underrepresented groups from being successful in a microbial profession while increasing diversity and inclusion (D&I) opportunities in their communities. It is also clear that establishing comprehensive strategic plans to address issues related to D&I, such as the ASM inclusive diversity with equity access and accountability (IDEAA) plan or the diversification of the journals’ editorial boards, is only the first step because other actions are needed; these will involve the entire microbial science community to guarantee the success of the proposed interventions.

It is important to understand that the microbial sciences are more effective when people from various backgrounds and with an array of perspectives come together to share a purpose and to achieve common goals. Here are some actionable points regarding areas of opportunity that need to be considered for the D&I initiatives to be effective and become agents of change in microbial communities.

## EDUCATE LEADERSHIP ABOUT D&I AND IDENTIFY TRUE ALLIES OR CHAMPIONS

Microbiologists, at one point during their training or later as part of their professional careers, can appreciate the value of education as a cornerstone for success. Implementation of D&I initiatives in any microbial science community begins with an educational program that spans from trainees to leaders, ensuring that every member understands the value of working or volunteering in an environment that empowers the D&I principles. This provides the tools to learn and grow and ensures that the programs can thrive in the respective community. Such training should include, but is not restricted to, topics on unconscious bias, diversity in the workplace, and how to identify and eliminate microaggressions toward underrepresented groups. Perhaps more importantly, this education should be continuous, providing leaders, particularly those overseeing decision-making and implementation of policies and procedures, with a full understanding of the value of D&I for the community at large that they serve. Additionally, these educated leaders must then pledge to become allies or champions for the D&I initiatives, thus empowering those in charge of the implementation. This empowerment will work to ensure that everybody understands the importance of such changes, and they can overcome the underutilization and underrepresentation of women, people of color, and other such historically underrepresented groups.

## THE RESPONSIBILITY OF CREATING A D&I ENVIRONMENT IS NOT THE EXCLUSIVE JOB OF THE UNDERREPRESENTED GROUPS

Maintaining a culture that embraces and promotes inclusive diversity is essential to any microbial science group. However, promotion and implementation of D&I initiatives is not the sole responsibility of the individual(s) in charge (i.e., director of diversity, chief diversity officer, D&I committee, etc.). Further, the members of underrepresented groups are not solely responsible for the D&I agenda of the microbial community. Individuals from those underutilized or underserved groups should be included and form partnerships with other members so that the initiatives are truly inclusive. Finally, D&I strategies should not be exclusively managed by the human resources group of an institution. D&I officers or committees should work together with human resources to examine the inclusiveness of D&I practices but also collaborate with other entities to ensure that the strategy is effectively applied to every person and program.

## INCLUDING UNDERREPRESENTED MEMBERS AT DIFFERENT LEVELS OF LEADERSHIP TO FULFILL A QUOTA IS A RECIPE FOR FAILURE

Microbiologists tend to look at data values and metrics as a measurement of the reproducibility and success of an experimental design. However, creating an atmosphere that welcomes D&I at all levels of the microbial sciences is more difficult than just adding an individual to a leadership group merely because they are a member of an underrepresented group and will fulfill a “quota.” A common mistake is to try to diversify the members, including the leaders, while leaving out inclusion. One can argue that diversifying a microbial science group will be ineffective if the members do not feel a valued part of the organization. Institutional values do not have color, race, gender, or other differences. Instead, the D&I values have to speak to the character of the group to build a culture of inclusion. Such inclusivity requires an environment for open and sometimes difficult conversations to help people understand about the D&I values and to have a clear commitment to train, educate, consult, and empower the underrepresented members in the decision-making of the organization.

## DECOLONIZING THE MICROBIAL SCIENCES INCLUDES CREATING A PATH FOR SUSTAINABLE LEADERSHIP OPPORTUNITIES FOR UNDERREPRESENTED OR UNDERUTILIZED GROUPS

It is not a secret that the microbial sciences have been disproportionately “colonized” by certain groups or individuals that have benefited from their leadership positions in order to advance their careers and empower them to make decisions that can affect an entire microbial community. However, it is also evident that to have a relevant community in the 21st century, it must be decolonized and repopulated with an inclusive culture and diverse group of individuals that can be part of the conversation to advance the microbial sciences. However, lack of diversity and inclusiveness cannot be fixed all at once, and therefore, change must be constant. The members of the microbial science community need to use the lessons from microbes to create a more balanced and homeostatic community. It is essential to encourage underserved or underrepresented individuals to engage in changes that can benefit the microbial community. Leaders must understand that creating a more diverse and inclusive environment will have an impact in the business model. Many studies have demonstrated the positive effects of diversity in the workplace and have also shown that D&I environments boost profitability, culture, and public perception. Every person in the microbial sciences needs to reflect on how they contribute to the maintenance of the power structures and how to decolonize the field for the well-being of individuals and society. Are microbiologists ready to lead the way on D&I initiatives and set an example for the rest of the professions and scientific fields?

